# Protective Effect of* Mangifera indica* Linn.,* Cocos nucifera* Linn., and* Averrhoa carambola* Linn. Extracts against Ultraviolet B-Induced Damage in Human Keratinocytes

**DOI:** 10.1155/2016/1684794

**Published:** 2016-03-09

**Authors:** Chalinee Ronpirin, Nattaporn Pattarachotanant, Tewin Tencomnao

**Affiliations:** ^1^Department of Preclinical Science, Faculty of Medicine, Thammasat University, Pathumthani 12120, Thailand; ^2^Department of Clinical Chemistry, Faculty of Allied Health Sciences, Chulalongkorn University, Bangkok 10330, Thailand

## Abstract

This study was aimed at investigating the antioxidant activity of* Mangifera indica* Linn.,* Cocos nucifera* Linn., and* Averrhoa carambola* Linn. and their biological effect on human keratinocytes affected by the ultraviolet B (UVB), a major cause of cell damage and skin cancer through induction of DNA damage, production of reactive oxygen species (ROS), and apoptosis. The richest antioxidant activity was found in ethanol fraction of* M. indica* (21.32 ± 0.66 mg QE/g dry weight), while the lowest one was found in aqueous fractions of* M. indica* and* C. nucifera* (1.76 ± 2.10 and 1.65 ± 0.38 mg QE/g dry weight, respectively). Ethanol and aqueous fractions of* A. carambola* (250 *µ*g/mL) significantly reduced the number of apoptotic cells. The expression of cleaved caspase 3 in UVB-treated group was significantly greater than that in untreated group. Both fractions of* A. carambola* (50, 100, and 250 *µ*g/mL) significantly decreased the expression of cleaved caspase 3. Regarding the induction of DNA repair, ethanol (100 and 250 *µ*g/mL) and aqueous (50, 100 and 250 *µ*g/mL) fractions of* A. carambola* significantly decreased the percentage of cyclobutane pyrimidine dimers (CPD). Taken together, our results suggest that both fractions of* A. carambola* may be potentially developed for dermal applications.

## 1. Introduction

Ultraviolet B (UVB) is a well-known risk factor playing a role in photoaging and skin cancer in epidermis through triggering DNA damage or generating reactive oxygen species (ROS). ROS are chemically reactive molecules containing oxygen and play the important roles in cell signaling and homeostasis. In environmental stress such as UV or heat exposure, ROS levels can dramatically increase causing cell structures and DNA damage and apoptosis [1, 2]. Prevention of UVB-induced damage in skin by lowering ROS production is an evidence-based strategy against photoaging and skin cancer.

Thailand is rich in fruits that are not only diversified but also inexpensive and delicious. Unfortunately, there have been a few researches with evidence-based findings that demonstrate the health benefits of these fruits. For example, resveratrol mostly found in grapes and red wine could exert photoprotective properties on UVB-irradiated cells. To reduce cell death in UVB-damaged skin, resveratrol reduced the production of ROS and attenuated the activation of caspase 3 and caspase 8 that play a major role in apoptosis [3]. Moreover, the extracts of* Elaeocarpus hygrophilus* (makoknum) and* Phyllanthus emblica* (makampom) had high antimicrobial and strong antioxidant activities [4].

Caspase 3 is an effector caspase protein frequently activated in mammalian cell apoptosis [5–7]. It is associated with the initiation of the death cascade. Pathways to caspase 3 activation are either extrinsic or intrinsic apoptotic pathways by interacting with caspase 8 and caspase 9, respectively. Besides apoptotic pathway, caspase 3 is essential for cell survival that converges on many events such as cell shrinkage, blebbing, chromatin condensation, and DNA fragmentation [7–10].

The other pathway through which UVB damages cells is DNA damage. UV induction of DNA damage is a factor that influences the normal life process of all organisms. Minor DNA damage is to allow effective repair, while more severe damage can induce apoptosis and cell cycle arrest. There are two types of UVB-induced DNA damage such as cyclobutane pyrimidine dimers (CPD) and pyrimidine (6-4) pyrimidone photoproducts (6-4PP). Two results (CPDs and 6-4PPs) are the transition of C to T and CC to TT. CPD contains a four-membered ring which is from the coupling of the double bonds (C=C) of pyrimidines. CPD is the major source of UV-induced mutations because these dimers interfere with base pairing during DNA replication. CPD is usually at a 5 to 10 folding higher frequency than 6-4PPs. In minor DNA damage, CPD is repaired by exogenous CPD photolyase [1, 2, 11].

Previous study provided the evidence to support the protective effect of Thailand native herb extracts on UVB-induced toxicity in human keratinocyte. It found that the extracts of turmeric and ginger could protect human keratinocyte from UVB-induced DNA damage and apoptosis through the attenuation of caspase 3 activity and CPD formation [12].

This objective of this study was to evaluate the protective effect of three Thai fruit species,* Mangifera indica* Linn.,* Cocos nucifera* Linn., and* Averrhoa carambola* Linn., on UVB-induced damage in human keratinocytes. All fruits selected in this investigation were evaluated for their antioxidant activities as potential mechanisms for antiapoptotic activity and induction of DNA repair in human keratinocyte cell line (HaCaT). Development of natural products for dermal applications is our future goal based on the findings of this work.

In traditional medicine,* M. indica* was used to clear digestion and acidity. It is antidiuretic, antidiarrheal, antiemetic, and cardiac herb. Its fruits are known as a potential source of natural antioxidants containing phenolic compounds, ascorbate, and *β*-carotene [13].

The aqueous extract of* C. nucifera* was found to contain a free amino acid, L-arginine, which reduced the free radical generation. Moreover, vitamin C significantly reduced lipid peroxidation and increased antioxidant enzymes.* C. nucifera* could reduce lipid peroxidation content due to the high content of L-arginine. Besides, the high content of polyphenol could maintain the normal levels of lipid in tissue and serum. The aqueous extract of* C. nucifera* may be a new source of antineoplastic and antimultidrug resistance activities [14].

*A. carambola* or star fruits contain high polyphenol contents which were contributed significantly in ferric reducing capacity and radical scavenging capacity. Their antioxidant capacities were significantly increased with ripening and associated with flavonol, flavones, and hydrolysable tannins [15].

## 2. Materials and Methods

### 2.1. Chemicals and Reagents

All reagents used in this study were of analytical grade. Dimethyl sulfoxide (DMSO) and ethanol were purchased from Merck (Darmstadt, Germany). 1,4-Dithiothreitol (DTT) was purchased from Bio Basic Inc. (Ontario, Canada). Phenylmethyl sulphonyl fluoride (PMSF) was purchased from United States Biochemicals (Cleveland, OH, USA). Kodak processing chemicals for autoradiography films, Amersham ECL Select Western blotting detection reagent, and Hyperfilm ECL were purchased from GE Healthcare (Piscataway, NJ, USA). Dulbecco's modified Eagle medium (DMEM)/high glucose were purchased from Sigma Aldrich Co. (St. Louis, MO, USA). Fetal bovine serum (FBS) and penicillin-streptomycin solution (10,000 units/mL of penicillin and 10,000 *μ*g/mL of streptomycin) were purchased from HyClone (Logan, UT, USA).

A solution of 30% acrylamide/bis-acrylamide (37.5 : 1) was purchased from Biorad (Hercules, CA, USA). Ammonium persulfate (APS) was purchased from EMD Millipore (Billerica, MA, USA). The monoclonal rabbit anticaspase 3 (8G10, cat#9665) and polyclonal rabbit anti-GAPDH (14C10, cat#2118) were purchased from Cell Signaling Technology (Beverly, MA, USA). FITC Annexin V Apoptosis Detection kit with PI was purchased from Biolegend (CA, USA). OxiSelect*™* Cellular UV-Induced DNA Damage ELISA Kit (CPD) was purchased from Cell Biolabs (CA, USA).

Folin Ciocalteu's phenol reagent, gallic acid, Aluminium Chloride (AlCl_3_), Sodium Acetate (NaOAc), and Sodium Carbonate (Na_2_CO_3_) were purchased from Sigma Aldrich (USA).

### 2.2. Cell Line

HaCaT cells, an immortalized human epidermal keratinocyte cell line, were purchased from cell line service (Heidelberg, Germany). They were cultured in DMEM/high glucose containing 10% FBS and antibiotics (100 U/mL penicillin and 100 *μ*g/mL streptomycin) at 37°C in a humidified atmosphere at 5% CO_2_.

### 2.3. Plant Materials

Thai fruits were collected from Pathumthani and Nakornpathom provinces. They were authenticated based on their characteristics by Professor Dr. Thaweesakdi Boonkerd (Department of Botany, Faculty of Science, Chulalongkorn University). The voucher specimens deposited at Professor Kasin Suvatabhandhu Herbarium (Department of Botany, Faculty of Science, Chulalongkorn University) were A015246 (BCU), A015247 (BCU), and A015251 (BCU) for* A. carambola*,* M. indica*, and* C. nucifera*, respectively.

### 2.4. Thai Fruit Extraction

The dried fruits were extracted by maceration method using absolute ethanol (ratio 1 : 2) at 4°C for 48 h and filtered. For extraction using water, the mixture (ratio 1 : 2) was incubated at 100°C for 30 min and filtered. The residues were extracted twice. The two filtrates were combined and concentrated by evaporation at 45°C. The crude extracts were dissolved in DMSO or kept at −80°C until further investigation.

### 2.5. Antioxidant Determination by Folin Ciocalteu Phenol Assay and Total Flavonoid of Determination

#### 2.5.1. Folin Ciocalteu Phenol Assay (FCP)

Thai fruit extracts (50 *µ*L) and 10% Folin Ciocalteu phenol reagent (50 *µ*L) were mixed and incubated in the dark at room temperature for 30 min. Na_2_CO_3_ solution (35 *µ*L) was added, mixed, and incubated in the dark at room temperature for 20 min. The absorbance of reaction was measured with a spectrophotometer at 750 nm. Gallic acid was used as a standard. The amount of phenolic compound is in gallic acid equivalent (GE) mg/g of dry weight.

#### 2.5.2. Detection of Total Flavonoid

Thai fruit extracts (50 *µ*L) were mixed with the solution (150 *µ*L of 100% ethanol, 10 *µ*L of 1 M NaOAc, and 10 *µ*L of AlCl_3_). The mixture was incubated in the dark at room temperature for 40 min and the absorbance was measured with a spectrophotometer at 415 nm. Quercetin was used as a standard. The content of flavonoid is in Quercetin equivalent (QE) mg/g of dry weight.

### 2.6. The Effect of Thai Fruits Extracts on Cell Viability by MTT Assay

Cells were seeded at 10,000 cells/well in 96-well plates and incubated at 37°C for 24 hours. Cells were treated with Thai fruit extracts at different concentrations ranging from 0 to 500 *µ*g/mL for 48 hours. MTT working solution was added at 20 *µ*L/well and incubated at 37°C for 4 hours. In this step, formazan product was formed. The cytotoxicity was detected by removing media carefully and dissolving formazan product with 150 *µ*L of 100% DMSO. Supernatant was collected by centrifuge and transferred to a new 96-well plate and the absorbance was measured at 550 nm. The percentage of cell viability was calculated by using the formula

(1) $$\begin{array}{c} \%\;cell\;\;viability=\frac{\left({Abs}_{treated\;\;cells}-{Abs}_{blank}\right)}{\left({Abs}_{untreated\;\;cells}-{Abs}_{blank}\right)}\times 100. \end{array}$$

### 2.7. The Effect of UVB on Cell Viability by Trypan Blue Exclusion

Cells were seeded in 6-well plates at 200,000 cells/well and cultured for 24 hours. Media were removed and cells were treated with UVB at different doses (0, 200, 400, 800, and 1600 mJ/cm^2^). After UVB treatment, media were added and cells were cultured for 24 hours. To determine cell viability Trypan blue dye (ratio 1 : 10) was used.

### 2.8. The effect of Thai Fruit Extracts on Cell Apoptosis by Flow Cytometer

Cells were seeded at 200,000 cells/well in 6-well plates and incubated at 37°C for 24 hours. Having been incubated, media were removed and cells were treated with UVB 200 mJ/cm^2^. After treatment, cells were treated with Thai fruit extracts at the concentrations of 50, 100, and 250 *µ*g/mL for 24 hours. Having been treated, media were removed and cold PBS was added to wash cells twice. Cells were harvested and recentrifuged at 400 g for 5 min. The supernatant was discarded and cells were resuspended in 100 *µ*L of 1x annexin-binding buffer. Cell suspension was added with 2.5 *µ*L of Alexa Fluor 488 annexin V and 5 *µ*L of PI and incubated at room temperature for 15 min. Having been incubated, 400 *µ*L of 1x annexin-binding buffer was added and cells were kept on ice. Cell apoptosis was analyzed by flow cytometry.

### 2.9. The Effect of Thai Fruit Extracts on Caspase 3 Protein Expression by Western Blotting

Cells were seeded at 200,000 cells/well in 6-well plates and incubated at 37°C for 24 hours. Having been incubated, media were removed and cells were treated with UVB 200 mJ/cm^2^. After treatment, Thai fruit extracts at the concentrations of 50, 100, and 250 *µ*g/mL were treated for 24 hours. In the following day, protein extraction was carried out using 1 mM of DTT and 1 mM of PMSF in NP-40 lysis buffer. Total protein (20 *μ*g) was mixed with Laemmli buffer (ratio 1 : 1) and boiled for 5 min. Protein was separated by 10% sodium dodecyl sulfate-polyacrylamide gel electrophoresis (SDS-PAGE) and transferred onto polyvinylidene difluoride (PVDF) membranes. Membranes were blocked with 5% nonfat milk either for 1 hour at room temperature or overnight at 4°C. Membranes were incubated with caspase 3 and GAPDH primary antibodies for 1 hour at room temperature or overnight at 4°C. After incubation, membranes were washed by 1x TBS-Tween 20 (TBST) for 15 min 3 times, incubated with secondary antibodies (anti-rabbit IgG, HRP-linked antibody) for 45 min at room temperature, and washed by TBST for 15 min 3 times. Protein bands were visualized by adding the enhanced chemiluminescence detection reagent and visualized by using Amersham Hyperfilm ECL and Kodak processing chemicals for autoradiography films. Each band was normalized against GAPDH as an internal control.

### 2.10. The Effect of Thai Fruit Extracts on UV-Induced DNA Damage

Cells were seeded at 200,000 cells/well in 6-well plates and incubated at 37°C for 24 hours. Having been incubated, media were removed and cells were treated with UVB 200 mJ/cm^2^. After treatment, cells were treated with Thai fruit extracts at the concentrations of 50, 100, and 250 *µ*g/mL for 24 hours.

#### 2.10.1. Fixation and Denaturation

Media were removed and 100 *µ*L of 75% methanol/25% acetic acid was added. Cells were incubated at room temperature for 30 min. Wells were aspirated and 100 *µ*L of 70% ethanol was added and then they were incubated at room temperature for 30 min. Wells were aspirated and 100 *µ*L of Denaturation Solution A was added and then they were incubated at room temperature for 5 min. Cells were gently washed with 200 *µ*L of Dulbecco's Phosphate-Buffered Saline (DPBS) containing magnesium and calcium. After aspirating wells, 100 *µ*L of Denaturation Solution B was added and then they were incubated at room temperature for 10 min. Wells were aspirated and 200 *µ*L of Assay Diluent was added and they were incubated at room temperature for 30 min.

#### 2.10.2. CPD Detection

100 *µ*L of diluted anti-CPD antibody was added to wells and they were incubated at room temperature for 1 hour on the orbital shaker. Wells were washed with 1x wash buffer.

### 2.11. Statistical Analysis

Data were presented as the mean ± standard error (SD). Means were from three or more independent experiments. Data were analyzed by one-way analysis of variance (one-way ANOVA), followed by* post hoc* Dunnett's test (*P* value < 0.05).

## 3. Results 

### 3.1. Total Phenol and Flavonoid Contents of* M. indica*,* C. nucifera*, and* A. carambola* Extracts

Results of phenol and flavonoid of* M. indica*,* C. nucifera*, and* A. carambola* extracts were shown in Table 1. In all assays, the richest antioxidant activity was found in ethanol fraction of* M. indica* (21.32 ± 0.66 mg QE/g dry weight by total flavonoid determination). The lowest antioxidant activities were found in aqueous fractions of both* M. indica* and* C. nucifera* (1.76 ± 2.10 and 1.65 ± 0.38 mg QE/g dry weight, resp.).

### 3.2. The Effect of of* M. indica*,* C. nucifera*, and* A. carambola* Extracts on Cell Viability

**Figure 1:**
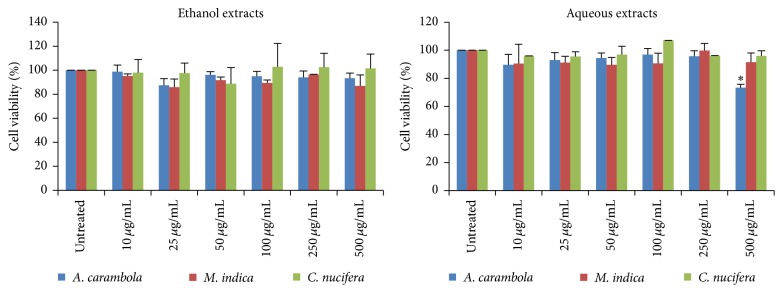
The effect of* M. indica*,* C. nucifera*, and* A. carambola* extracts on viability of HaCaT cells. Detection of cell viability was performed using MTT assay. HaCaT cells were treated with Thai fruit extracts at the concentration of 0–500 *µ*g/mL for 48 h. The aqueous extract of* A. carambola* could significantly decrease cell viability. *∗* The aqueous extract of* A. carambola* at the concentration of 500 *µ*g/mL could significantly decrease cell viability in comparison with the cell viability of untreated group.

To evaluate the effect of Thai fruit extracts on HaCaT cell viability, MTT assay was employed. Cell was treated with the different concentrations of extracts (0–500 *µ*g/mL). Results of cytotoxicity of all extracts were shown in Figure 1. The aqueous extract of* A. carambola* at the concentration of 500 *µ*g/mL could significantly decrease cell viability (73.42% ± 3.66, *P* < 0.05). Therefore, three concentrations of all extracts used in this study were 50, 100, and 250 *µ*g/mL.

### 3.3. The Effect of UVB Intensity on Cell Viability

**Figure 2:**
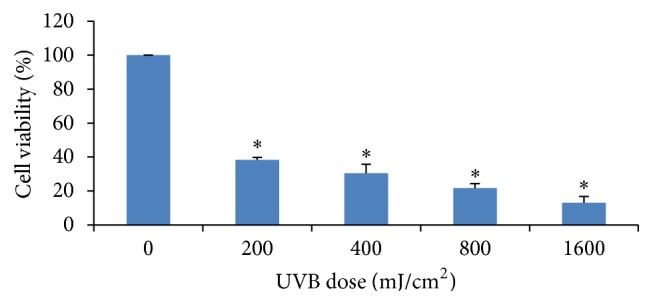
The effect of all UVB intensities on cell viability by Trypan blue assay. Cell viability values shown as mean ± SD were derived from 3 independent experiments. *∗* The cell viability in UVB-treated groups (200-1,600 mJ/cm^2^) significantly decreased when comparing to that in UVB-untreated group (0 mJ/cm^2^).

Evaluating the effect of UVB intensity on cell viability was employed by Trypan blue assay. Cells were treated with different intensities of UVB (0–1,600 mJ/cm^2^). The lowest intensity that could significantly decrease cell viability was 200 mJ/cm^2^. Results of the effect of all intensity of UVB on cell viability were shown in Figure 2.

### 3.4. The Protective Effect of All Fractions of* M. indica*,* C. nucifera*, and* A. carambola* Extracts on UVB-Induced Apoptosis by Flow Cytometry

**Figure 3:**
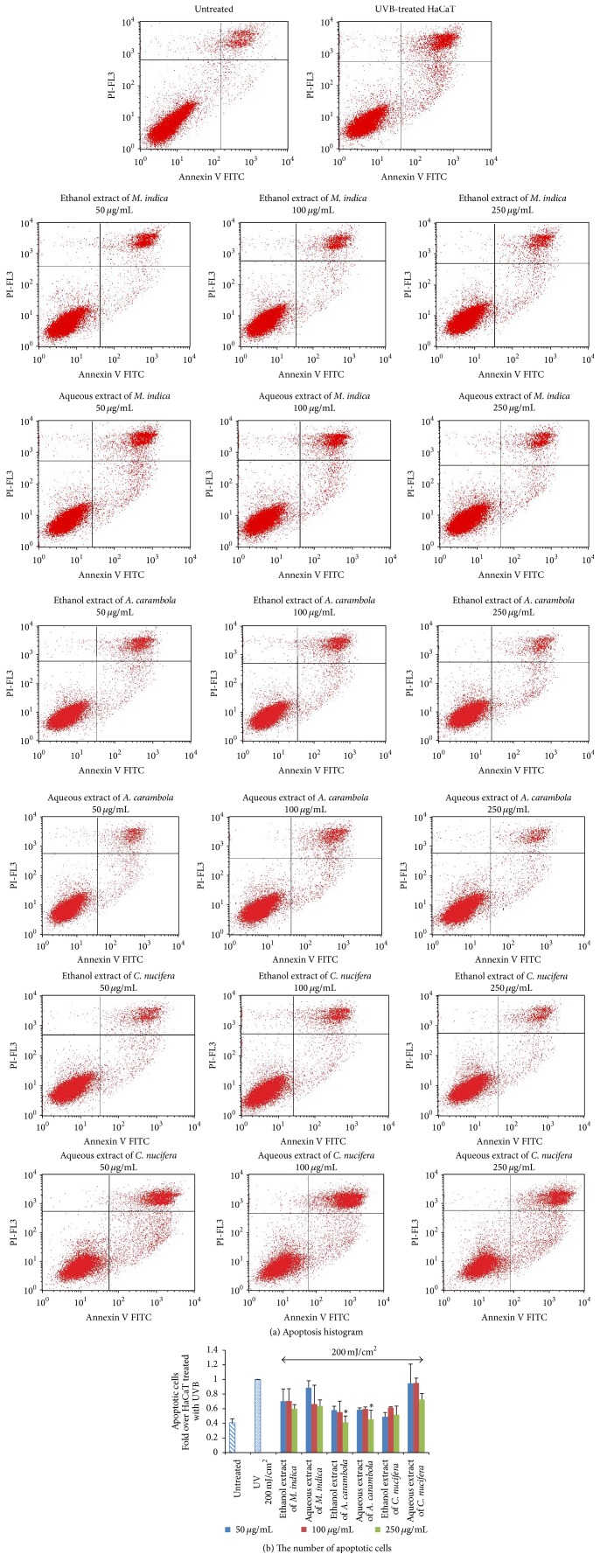
The effect of* M. indica*,* C. nucifera*, and* A. carambola* extracts on apoptosis of UVB-treated HaCaT cells. HaCaT cells were treated with UVB intensity at 200 mJ/cm^2^ and Thai fruit extracts at the concentration of 50, 100, and 250 *µ*g/mL for 24 h. Apoptotic cell images were shown as histogram (a) and apoptotic values were shown as mean ± SD derived from 3 independent experiments (b). *∗* The extracts of both fractions of* A. carambola* (250 *µ*g/mL) could significantly decrease the number of apoptotic cells in comparison with the number of apoptotic cells in the UVB-treated group.

Results of the protective effect of all fractions on UVB-induced apoptosis were shown in Figures 3(a) and 3(b). Ethanol and aqueous fractions of* A. carambola* (250 *µ*g/mL) could significantly decrease the number of apoptotic cells in comparison with the number of apoptotic cells in the UVB-treated group (*P* < 0.05).

### 3.5. The Effect of* M. indica*,* C. nucifera*, and* A. carambola* Extracts on Caspase 3 Expression by Western Blot

**Figure 4:**
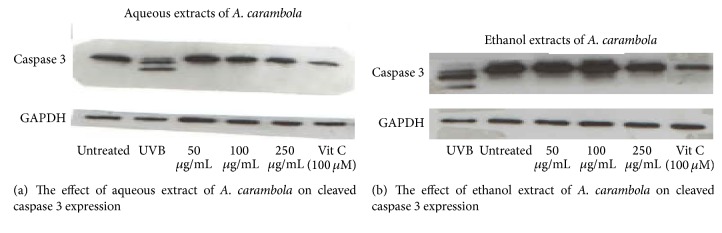
The effect of both aqueous (a) and ethanol (b) extracts of* A. carambola* on cleaved caspase 3 expression. Cleaved caspase 3 expression was increased when cells were treated with UVB (200 mJ/cm^2^). After UVB stimulation, cells were treated with extracts for 24 h. Both extracts of* A. carambola* could significantly decrease cleaved caspase 3 expression.

Since caspase 3 plays a major role in caspase-dependent apoptosis, the effect of Thai fruit extracts on the reduction of cleaved caspase 3 expression was investigated in this investigation. Using Western blot analysis, the aqueous extract (Figure 4(a)) and the ethanol extract (Figure 4(b)) of* A. carambola* could decrease the cleavage of caspase 3 expression after 24 hours of extract treatment. Vitamin C was used as a standard.

### 3.6. The Effect of* M. indica*,* C. nucifera*, and* A. carambola* Extracts on the Induction of DNA Repair by Cyclobutane Pyrimidine Dimers (CPD) Detection

**Figure 5:**
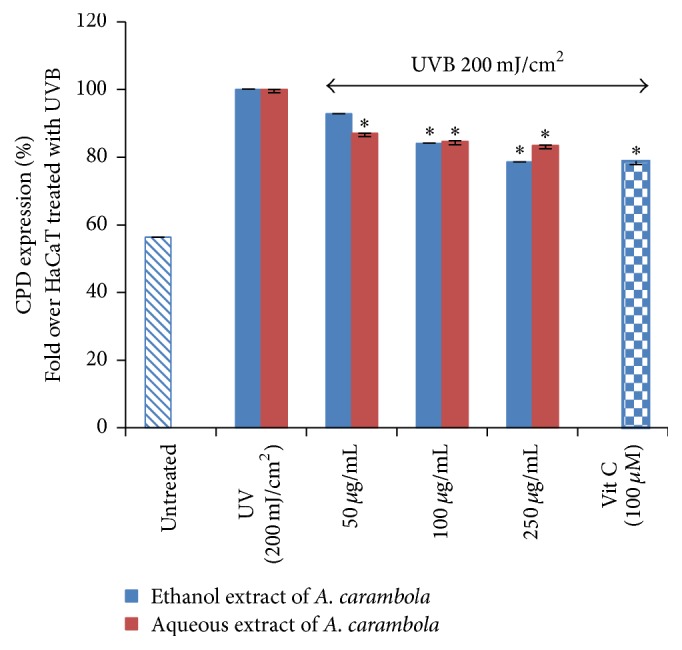
CPD expression in cells treated with UVB 200 mJ/cm^2^; both extracts of* A. carambola* and 100 *µ*M of vitamin C. The level of CPD expression was low in untreated group. UVB could induce DNA damage causing CPD expression to increase. After extract treatment for 24 h, CPD expression was significantly decreased. Values of CPD expression were shown as mean ± SD derived from 3 independent experiments. *∗* The ethanol (100 and 250 *µ*g/mL) and aqueous (50, 100, and 250 *µ*g/mL) fractions of* A. carambola* could significantly decrease the percentage of CPD in comparison with the percentage of CPD in the UVB-treated group.

CPD is the product of UVB-induced DNA lesions. In this study, UVB (200 mJ/cm^2^) treatment could significantly increase CPD expression. After the treatment of Thai fruit extracts, the result showed that ethanol (100 and 250 *µ*g/mL) and aqueous (50, 100, and 250 *µ*g/mL) fractions of* A. carambola* could significantly decrease the percentage of CPD (*P* < 0.05). The results were shown in Figure 5.

## 4. Discussion

UVB is a major cause of cell damage and skin cancer through inducing DNA damage and apoptosis. There are two pathways that decrease UVB-induced cell damage such as antiapoptosis and DNA damage repair. The effect of these extracts on apoptosis was detected by flow cytometry and Western blot analysis.

According to the flow cytometric data, the percentage of apoptotic cells in the untreated group was significantly different from that in the UVB-treated group, suggesting that UVB at 200 mJ/cm^2^ could lead to the increase in apoptotic cells. In addition, both ethanol and aqueous extracts of* A. carambola* at the concentration of 250 *µ*g/mL could significantly decrease the percentage of the number of apoptotic cells (*P* < 0.05).

To confirm the effect of both extracts on protecting UV-induced apoptosis, the expression of caspase 3 was detected by Western blot. Many studies indicated that caspase 3 (35 kDa) in UVB-treated cells was cleaved. Cleaved caspase 3 (17 and 19 kDa) is an important factor which plays a role in the induction of cell apoptosis through apoptotic pathway [7, 16–21].

It was recently reported that vitamin C exerted antiapoptotic activity by attenuating caspase 3 expression [22, 23]. Therefore, vitamin C was used as a control in this study. Results of the expression of cleaved caspase 3 decreasing in both ethanol and aqueous fractions of* A. carambola*-treated and vitamin C-treated cells implied that the attenuation of cleaved caspase 3 was involved in cell survival after UVB irradiation [20, 24, 25].

The results showed that the level of CPD expression was increased when treated with UVB. After UVB radiation, CPD level in* A. carambola*-treated group was significantly decreased (*P* < 0.05).

The extract of* A. carambola* has been used in the traditional medicine for treating many diseases such as diabetes and diabetic nephropathy. Many studies indicated that it could inhibit apoptotic pathway by attenuating the activation of caspase 3, caspase 8, and caspase 9 [26, 27]. Level of active caspase 3 can affect the formation of DNA fragmentation, since caspase 3 is a primary activator which induces the cleavage of DNA fragmentation factor (DFF) complex. Cleaved DFF causes DNA damage and cell death [9, 28]. To date, there are not many studies to investigate the effect of* A. carambola* extract on DNA damage and cytotoxicity.

Collectively, our results showed that both ethanol and aqueous fractions of* A. carambola* could attenuate UVB-induced damage in human keratinocytes by inhibiting the cleavage of caspase 3 and CPD formation in the HaCaT keratinocyte cell line. The present study is the first to provide the evidence of potent protective effect of* A. carambola* extract against ultraviolet B-induced damage in human keratinocytes. The extracts of* A. carambola* may be developed as the agent for the protection of UVB-induced damage in skin.

## Figures and Tables

**Table 1:** Total phenol and flavonoid contents of *M. indica*, *C. nucifera, *and* A. carambola* extracts derived from ethanol and aqueous solvents.

Extracts	Phenol content		Flavonoid content	
	(mg GE/g dry weight)		(mg QE/g dry weight)	
	Ethanol	Aqueous	Ethanol	Aqueous
*M. indica*	3.04 ± 2.52	3.22 ± 0.11	21.32 ± 0.66	1.76 ± 2.10
*A. carambola*	4.13 ± 1.51	5.27 ± 2.96	6.34 ± 0.13	5.94 ± 0.60
*C. nucifera*	2.21 ± 0.11	4.36 ± 0.12	2.89 ± 1.89	1.65 ± 0.38

## References

[1] Rastogi R. P., Richa, Kumar A., Tyagi M. B., Sinha R. P. (2010). Molecular mechanisms of ultraviolet radiation-induced DNA damage and repair. *Journal of Nucleic Acids*.

[2] Lo H.-L., Nakajima S., Ma L. (2005). Differential biologic effects of CPD and 6-4PP UV-induced DNA damage on the induction of apoptosis and cell-cycle arrest. *BMC Cancer*.

[3] Narayanan D. L., Saladi R. N., Fox J. L. (2010). Review: ultraviolet radiation and skin cancer. *International Journal of Dermatology*.

[4] Nanasombat S., Khanha K., Phan-im J. (2012). Antimicrobial and antioxidant activities of Thai local fruit extracts: application of a selected fruit extract. *Phyllanthus emblica* Linn. as a natural preservative in raw ground pork during refrigerated storage. *The Online Journal of Science and Technology*.

[5] Nicholson D. W., Thornberry N. A. (1997). Caspases: killer proteases. *Trends in Biochemical Sciences*.

[6] Jacobson M. D., Weil M., Raff M. C. (1997). Review: programmed cell death in animal development. *Cell*.

[7] Porter A. G., Jänicke R. U. (1999). Review: emerging roles of caspase-3 in apoptosis. *Cell Death & Differentiation*.

[8] Jänicke R. U., Sprengart M. L., Wati M. R., Porter A. G. (1998). Caspase-3 is required for DNA fragmentation and morphological changes associated with apoptosis. *The Journal of Biological Chemistry*.

[9] Wolf B. B., Schuler M., Echeverri F., Green D. R. (1999). Caspase-3 is the primary activator of apoptotic DNA fragmentation via DNA fragmentation factor-45/inhibitor of caspase-activated DNase inactivation. *The Journal of Biological Chemistry*.

[10] Sahara S., Aoto M., Eguchi Y., Imamoto N., Yoneda Y., Tsujimoto Y. (1999). Acinus is a caspase-3-activated protein required for apoptotic chromatin condensation. *Nature*.

[11] Drouin R., Therrien J.-P. (1997). UVB-induced cyclobutane pyrimidine dimer frequency correlates with skin cancer mutational hotspots in p53. *Photochemistry and Photobiology*.

[12] Thongrakard V., Ruangrungsi N., Ekkapongpisit M., Isidoro C., Tencomnao T. (2014). Protection from UVB toxicity in human keratinocytes by thailand native herbs extracts. *Photochemistry and Photobiology*.

[13] Rocha Ribeiro S. M., De Queiroz J. H., Lopes Ribeiro de Queiroz M. E., Campos F. M., Pinheiro Sant'Ana H. M. (2007). Antioxidant in mango (*Mangifera indica* L.) pulp. *Plant Foods for Human Nutrition*.

[14] DebMandal M., Mandal S. (2011). Coconut (*Cocos nucifera* L.: Arecaceae): in health promotion and disease prevention. *Asian Pacific Journal of Tropical Medicine*.

[15] Lim Y. S., Lee S. T. (2013). In vitro antioxidant capacities of star fruit (*Averrhoa carambola*), an underutilised tropical fruit. *Journal of Biology*.

[16] Lee C.-H., Wu S.-B., Hong C.-H., Yu H.-S., Wei Y.-H. (2013). Molecular mechanisms of UV-induced apoptosis and its effects on skin residential cells: the implication in UV-based phototherapy. *International Journal of Molecular Sciences*.

[17] Park K., Lee J.-H. (2007). Photosensitizer effect of curcumin on UVB-irradiated HaCaT cells through activation of caspase pathways. *Oncology Reports*.

[18] Namura S., Zhu J., Fink K. (1998). Activation and cleavage of caspase-3 in apoptosis induced by experimental cerebral ischemia. *Journal of Neuroscience*.

[19] Roy A. M., Baliga M. S., Elmets C. A., Katiyar S. K. (2005). Grape seed proanthocyanidins induce apoptosis through p53, bax, and caspase 3 pathways. *Neoplasia*.

[20] Mendelev N., Witherspoon S., Li P. A. (2009). Overexpression of human selenoprotein H in neuronal cells ameliorates ultraviolet irradiation-induced damage by modulating cell signaling pathways. *Experimental Neurology*.

[21] Park Y.-K., Jang B.-C. (2014). UVB-induced anti-survival and pro-apoptotic effects on HaCaT human keratinocytes via caspase- and PKC-dependent downregulation of PKB, HIAP-1, Mcl-1, XIAP and ER stress. *International Journal of Molecular Medicine*.

[22] Cheng B., Zhang Y., Wang A., Dong Y., Xie Z. (2014). Vitamin C attenuates isoflurane-induced caspase-3 activation and cognitive impairment. *Molecular Neurobiology*.

[23] Ozmen O., Mor F. (2014). Effects of vitamin C on pathology and caspase-3 activity of kidneys with subacute endosulfan toxicity. *Biotechnic & Histochemistry*.

[24] Park K., Lee J.-H. (2008). Protective effects of resveratrol on UVB-irradiated HaCaT cells through attenuation of the caspase pathway. *Oncology Reports*.

[25] Jing L., Kumari S., Mendelev N., Li P. A. (2011). Coenzyme Q10 ameliorates ultraviolet B irradiation induced cell death through inhibition of mitochondrial intrinsic cell death pathway. *International Journal of Molecular Sciences*.

[26] Wen Q., Liang T., Qin F. (2013). Lyoniresinol 3*α*-O-*β*-D-glucopyranoside-mediated hypoglycaemia and its influence on apoptosis-regulatory protein expression in the injured kidneys of streptozotocin-induced mice. *PLoS ONE*.

[27] Xu X., Liang T., Wen Q. (2014). Protective effects of total extracts of *Averrhoa carambola* l. (oxalidaceae) roots on streptozotocin-induced diabetic mice. *Cellular Physiology and Biochemistry*.

[28] Sharif-Askari E., Alam A., Rhéaume E. (2001). Direct cleavage of the human DNA fragmentation factor-45 by granzyme B induces caspase-activated DNase release and DNA fragmentation. *The EMBO Journal*.

